# Orofacial myofunctional signs and symptoms in adults with sleep breathing disorder: is there a correlation?

**DOI:** 10.1590/2317-1782/e20240033en

**Published:** 2025-04-28

**Authors:** Gabriele Ramos de Luccas, Raphaela Godoi Abu Halawa, Carlos Henrique Ferreira Martins, Giédre Berretin-Felix

**Affiliations:** 1 Departamento de Fonoaudiologia, Faculdade de Odontologia de Bauru – FOB, Universidade de São Paulo – USP - Bauru (SP), Brasil.

**Keywords:** Obstructive Sleep Apnea, Snoring, Speech, Language and Hearing Sciences, Myofunctional Therapy, Tongue, Stomatognathic System

## Abstract

**Purpose:**

to verify whether orofacial myofunctional symptoms are related to findings from orofacial myofunctional clinical assessment in adults sleeping breathing disorders (SBD).

**Methods:**

observational study; 15 adults, with a mean age of 43 years and diagnosed with primary snoring or OSA by polysomnography; evaluated using the Orofacial Myofunctional Assessment Protocol MBGR, including the Clinical **History** Protocol to assess symptoms, and the Clinical Examination Protocol to identify signs, considering tests of mobility of lips, tongue, soft palate and jaw; tone of lips, tongue, cheeks and chin; respiratory mode; chewing; and swallowing solids and liquids. For the correlation between signs and symptoms, Spearman's Correlation Coefficient, considering p<0.05 statistically significant, was used.

**Results:**

The main orofacial myofunctional complaints were related to chewing (use of only one side during chewing and the need to drink liquids during meals), and swallowing (choking and residue after swallowing). In the orofacial myofunctional assessment, there was a greater frequency of alterations in tongue tone; lip mobility; unilateral chewing pattern with increased speed and chewing inefficiency; swallowing with excessive contraction of the perioral muscles, associated head movement and presence of residue in the oral cavity. The correlation between the scores of orofacial myofunctional signs and symptoms showed significance only between the aspects related to the chewing function (p=0.034), being moderate and inversely proportional (r=-0.548).

**Conclusion:**

a moderate negative correlation was found between masticatory signs and symptoms in adults with SBD, and no correlation was observed for breathing and swallowing functions.

## INTRODUCTION

Sleep-disordered breathing (SDB) is a chronic multifactorial condition with consequences related to sleep quality, daytime symptoms of sleepiness and impairment of daily activities^(1)^. The most common SDB are primary snoring and obstructive sleep apnea (OSA), which refer to episodes of upper airway obstruction during sleep. In cases of OSA, there is an association with several comorbidities, such as cardiovascular and metabolic diseases^(2)^.

Early diagnosis is essential and is obtained mainly through polysomnography exams. The treatment is interdisciplinary and involves the participation of doctors, physiotherapists, dentists, speech-language pathologists, among other professionals, to discuss and define the best therapeutic strategies, which include isolated or combined weight loss treatment, use of Continuous Positive Airway Pressure (CPAP), mandibular advancement devices, surgeries^(3)^, orofacial myofunctional therapy (OMT)^(4)^, among other approaches.

Studies on the results of OMT for cases of primary snoring and OSA began to be developed in Brazil in 2009, with treatment proposals that included oropharyngeal exercises and intervention in orofacial functions^(5)^. According to the literature, there is currently data demonstrating the effectiveness of this treatment in reducing signs and symptoms of OSA and the intensity and frequency of snoring, in addition to improving orofacial myofunctional aspects^(6-8)^.

The identification of orofacial myofunctional alterations presented by patients with OSA was the initial starting point for the development of treatment proposals within the scope of the Orofacial Motricity area, in a speech-language pathology therapy context. In general, these individuals tend to have an elongated soft palate, altered lingual muscles, sagging uvula, palatoglossal arch, facial mimic muscles, lateral pharyngeal wall, buccinator, orbicularis oris and suprahyoid muscles, little facial mobility, changes in chewing and swallowing, and oronasal breathing^(9)^.

Orofacial myofunctional therapeutic planning for SDB cases is based on scientific evidence and clinically personalized for each patient, considering their particularities and main myofunctional alterations and limitations. In this sense, the orofacial myofunctional evaluation process is essential, as it allows the analysis and interpretation of important structural, muscular and functional aspects that must be taken into consideration in therapeutic objectives and goals.

Clinical protocols of orofacial myofunctional assessment are applied in OMT research for cases of SDB with the aim of analyzing pre- and post-intervention results and, although they address the orofacial myofunctional characteristics of this population^(10,11)^, there are still no more detailed studies on these characteristics, especially those that correlate clinical data with patient symptoms and complaints.

Orofacial myofunctional symptoms may include complaints related to the orofacial functions of breathing, chewing, swallowing and speaking, and may also indicate problems related to facial aesthetics. In the clinical context, identifying symptoms at the beginning of the therapeutic process is essential to outline treatment priorities and align expectations with patients. Likewise, the identification of clinical signs is important for directing and selecting exercises and therapeutic strategies. The relationship between the presence of signs and symptoms is common, but there may be cases in which the patient has no self-perception of their myofunctional changes, which results in the absence of complaints and symptoms.

Obtaining data on the main orofacial myofunctional signs and symptoms presented by patients with SDB, as well as the correlation between this information, can help direct the perspective of the clinical speech-language pathology therapist who serves this population. Considering that such information can improve theoretical-practical reasoning, the present study aims to verify whether orofacial myofunctional symptoms are related to findings of the orofacial myofunctional clinical evaluation in adults with SDB.

## METHODS

This is an observational study previously approved by the Research Ethics Committee of the Bauru School of Dentistry of the University of São Paulo (FOB/USP) (CAAE: 53781916.7.0000.5417), statement no. 3.284.811. All research participants were included in the study by signing an Informed Consent Form (ICF).

Fifteen adults with a mean age of 43 years (standard deviation: 10.2) and a diagnosis of primary snoring or OSA, as evidenced by a polysomnography exam reported by a sleep doctor specialist, were selected. Participants were recruited through medical referrals to the Speech-Language Pathology and Audiology Therapy Clinic of FOB/USP. Data collection and analysis procedures were also performed at the respective clinic from July to December 2016 by a single researcher.

Participants aged between 19 and 59 years of both genders and diagnosed with OSA of varying degrees or primary snoring confirmed by polysomnography were included in the study. Adults with a history of lung diseases, such as acute respiratory failure, pulmonary embolism, asthma, need for ventilatory support, post-thoracic surgery, neurological disorders, oncological diseases of the head and neck, current smokers, previous speech-language pathology therapy in the area of ​​orofacial motricity or oropharyngeal dysphagia, and a recent polysomnography exam performed more than six months ago, were excluded.

The inclusion and exclusion criteria were applied based on an initial interview to collect clinical history and previous orofacial myofunctional assessment, based on a clinical examination protocol^(10)^, considering facial pattern analysis tests and intraoral analysis to exclude individuals with lingual frenulum alterations, malocclusion, and maxillomandibular discrepancies.

After selecting the research sample, all study participants underwent the same evaluation and analysis procedures.

### Orofacial Myofunctional Assessment Protocol MBGR

The Orofacial Myofunctional Assessment Protocol MBGR is an assessment instrument with scores consisting of clinical history and clinical examination, developed for speech-language pathology professionals who work in the Orofacial Motricity area^(10)^. To apply this protocol in clinical practice or research, adequate training is required for correct data collection and interpretation.

The researcher responsible for the study completed courses and practical sessions to apply the instrument in a standardized manner to model patients, under the direct supervision of one of the authors of the protocol. The researcher performed all procedures on the patients in this study.

### Clinical History – MBGR

The protocol for collecting clinical history data consists of questions that seek to identify complaints and symptoms related to health problems, previous treatments, sleep, breathing problems, chewing, swallowing, current diet, oral habits, and also aspects involving communication, speech, hearing, voice and education. In order to facilitate the tabulation and correlation of data, the researchers stipulated a score for each item of the protocol according to the frequency of the symptom presented by the patient, being 0 (absence of symptom), 1 (sporadic presence of symptoms), and 2 (frequent presence of symptoms). Therefore, the higher the score, the greater the patient's complaint regarding that aspect.

The researcher conducted an interview with the patients in a comfortable and quiet place to collect and record the information provided.

### Orofacial myofunctional clinical examination

In addition to the clinical history, the protocol contains a proposal for assessing orofacial myofunctional aspects called MBGR Orofacial Myofunctional Evaluation, which assigns scores to classify the alterations found. In this sample, the protocol included the aspects described below, with each item being assigned a value, with the number 0 representing the absence of alteration and higher numbers representing the presence of alteration, and the higher the total value, the greater the level of alteration. The following items of the protocol were performed with the patients:

Lip, tongue, soft palate and jaw mobility: a verbal request was made for motor tasks, which were filmed for later analysis. To assess lip mobility, the following movements were analyzed: protruding closed and open, retracting closed and open, protruding closed to the right and left, and snapping protracted and retracted. The tongue was assessed by protruding and retracting, raising the tip on the incisive papilla, cardinal points with the tip of the tongue, touching the right and left cheeks internally, snapping the tip and body, sucking the tongue on the palate and vibrating. The soft palate was assessed by repeatedly emitting the vowel [a] and the jaw was analyzed by opening and closing the mouth, laterality to the right and left, observing the presence of deviation and/or pain;
Tonicity of lips, tongue, cheeks and chin: the muscles were palpated so that they could be classified as normal, hypotonic or hypertonic;
Breathing mode: the classification was performed as nasal, oronasal or oral. The Glatzel millimeter mirror was used to check the patient's nasal airflow upon arrival and after cleaning. The symmetry of the flow between the nostrils was observed, in addition to evaluating the possibility of nasal use with the mouth water test, in which the patient was asked to keep the liquid in the oral cavity for two minutes or more to be considered adequate;
Chewing: the usual chewing of three portions of wafer biscuits was evaluated, observing the incision, grinding, time, chewing pattern, lip closure, speed, noises and atypical muscle contractions;
Swallowing: habitual and directed swallowing of liquids (water at room temperature offered in a transparent glass) and solids (wafer biscuits) was assessed. Lip closure, tongue posture, restraint, atypical muscle contractions, head movement, noises, coordination and residues after swallowing were observed.

### Data analysis

Descriptive analysis was used to present sample characterization data. Spearman's correlation coefficient was used to correlate signs and symptoms, considering p<0.05 as statistically significant. The total scores of signs and symptoms were considered for correlation, as well as specific items of the Clinical History Protocol (health problems; breathing problems; sleep, oral habits; chewing; swallowing) that were correlated with items of the Protocol of Orofacial Myofunctional Evaluation (mobility, tonicity, breathing, chewing, swallowing). The items of sleep symptoms and oral habits were correlated only with the signs of orofacial functions (breathing, chewing and swallowing).

The correlation strength obtained was classified based on the reference of a previous study^(12)^, in which r=0.10 to 0.30 is considered weak; r=0.40 to 0.60 is moderate; and =0.70 to 1 is strong.

## RESULTS

Initially, 38 individuals diagnosed with SDB were considered for this study. Of these, 14 refused to participate due to the inability to attend the appointments; two did not attend the appointments scheduled for data collection; and seven did not meet the inclusion and exclusion criteria due to ankyloglossia (n=4), facial pattern II (n=2) and absence of teeth (n=1).

Table 1 shows the data characterizing the participants, such as age, sex, Apnea Hypopnea Index (AHI) and total score of symptoms and signs obtained by the MBGR Protocol. The sample consisted of 15 participants, the majority of whom were male (73.3%), with a predominant age range between 40 and 50 years and with varying degrees of OSA severity. The scores for signs and symptoms varied among the participants, with 18 and 45 being the minimum and maximum scores for symptoms and 15 and 39 for signs, respectively.

**Table 1:** Sample characterization regarding sex, age, Apnea-Hypopnea Index (AHI), and total score of symptoms and signs obtained by the MBGR Protocol

Patient	Sex	Age	AHI	Total Symptom Score	Total Sign Score
				Min: 0	Min: 0
				Max: 216	Max: 175
1	F	38	3.3	41	31
2	M	55	67.7	28	33
3	M	43	2.8	35	21
4	M	37	24.3	30	15
5	F	22	6.8	42	17
6	M	34	5.8	36	27
7	M	59	19.4	29	28
8	F	50	7.0	29	39
9	M	43	57.4	35	26
10	M	42	14.7	38	26
11	M	49	4.4	43	34
12	M	56	14	25	35
13	F	31	6.8	59	21
14	M	34	7.9	45	16
15	M	49	29.1	18	22

**Caption: F =** Female; **M =** Male; **AHI =** Apnea-Hypopnea Index

Table 2 shows the scores obtained for each participant about health problems, breathing problems, chewing, swallowing, sleep, and oral habits. It can be seen that the highest scores refer to sleep complaints, with patients reporting snoring (n=14), heavy breathing during sleep (n=11) and sleep apnea (n=13) more frequently.

**Table 2:** General aspects of the sample regarding complaints related to health problems, respiratory problems, chewing, swallowing, sleep, and oral habits

Patient	Health Issues	Respiratory Problems	Chewing	Swallowing	Sleep	Oral Habits
	Min: 0	Min: 0	Min: 0	Min: 0	Min: 0	Min: 0
	Max: 5	Max: 13	Max: 16	Max: 22	Max: 22	Max: 5
1	0	6	3	4	7	2
2	2	1	2	0	13	2
3	2	0	1	2	14	0
4	1	1	2	0	15	2
5	2	4	2	0	17	2
6	1	4	1	1	8	3
7	2	3	2	0	14	2
8	2	5	0	1	9	0
9	1	3	7	0	11	0
10	0	7	1	1	10	1
11	1	1	2	0	12	3
12	1	3	3	1	7	2
13	4	1	3	3	17	3
14	1	6	3	2	10	3
15	1	4	0	3	1	1

The health problems reported were orthopedic (n=7), metabolic (n=7), digestive (n=2) and hormonal (n=2), while breathing problems referred to nasal obstruction (n=8), rhinitis (n=8), runny nose (n=7), halitosis (n=5) and nasal itching (n=5). Regarding biting habits, these indicated, in their majority, clenching of teeth (n=10), bruxism (n=7) and the habit of biting the mucosa (n=5). As for chewing, the main complaints presented were related to the use of only one side during chewing (n=11) and the need to drink liquids during meals (n=6). As for swallowing, the complaints presented were related to choking (n=6), residues after swallowing (n=3) and difficulty swallowing (n=1).

Table 3 presents the individual results of the orofacial myofunctional assessment performed using the MBGR protocol, which includes the scores obtained for tonicity (lips, tongue, cheeks and chin), mobility (lips, tongue, soft palate and jaw), breathing, chewing and swallowing.

**Table 3:** Presentation of individual scores obtained for study participants in the tonicity, mobility, breathing, chewing and swallowing tests in the clinical assessment

Patient	Tonicity	Mobility	Breathing	Chewing	Swallowing
N: 15	Min: 0	Min: 0	Min: 0	Min: 0	Min: 0
	Max: 6	Max: 68	Max: 4	Max: 10	Max: 36
1	5	14	0	2	9
2	2	18	0	2	10
3	2	4	0	5	9
4	2	5	0	2	6
5	5	7	0	2	2
6	4	14	0	3	5
7	4	13	0	3	7
8	3	26	0	3	6
9	4	17	0	2	2
10	4	7	0	3	11
11	3	15	0	1	14
12	3	19	0	2	10
13	1	9	0	2	8
14	1	6	0	2	7
15	5	7	0	2	7

Concerning orofacial tonicity, there was a higher frequency of hypotonia for the tongue (n=12), followed by lips (n=9) and cheeks (n=7), and no participant presented increased tone.

About mobility, alterations in lip mobility were the most frequent (n=11). For all mobility tests, patients presented scores indicating normality or alteration, and no inability to perform any requested movement was found. It was also frequently noted that most patients presented associated movements of the cervical muscles when performing the tongue and jaw mobility tests.

As for breathing function, all patients presented nasal breathing mode (n=15). In masticatory function, the alterations found refer to unilateral masticatory pattern (n=7), increased speed (n=9) and masticatory inefficiency (n=8). Finally, in swallowing, most alterations are related to excessive contraction of the perioral muscles (n=11), associated head movement (n=4) and presence of residues in the oral cavity (n=5).

Table 4 shows that there was a significant correlation between the scores of orofacial myofunctional signs and symptoms only between the aspects related to the chewing function (p=0.034), being inversely proportional and with moderate correlation strength (r=-0.548).

**Table 4:** Presentation of the correlations obtained between signs and symptoms of breathing, chewing, and swallowing functions.

Total Symptom Score	Total Sign Score	Value r	Value p
Breathing	Breathing	-0.046	0.870
Chewing	Chewing	-0.548	0.034*
Swallowing	Swallowing	0.191	0.496

* p<0.05

## DISCUSSION

The orofacial myofunctional characteristics of patients with SDB guide speech-language pathologists in the selection of individuals eligible for treatment, in addition to being fundamental for the choice and selection of personalized therapeutic goals and strategies for each case. The MBGR Protocol is frequently used in the clinical evaluation of patients with SDB in the context of randomized clinical trials^(5,6)^, in addition to being frequently used in the clinical routine of speech-language pathology therapists, consisting of a survey of clinical complaints and orofacial myofunctional evaluation^(10)^.

The survey of clinical complaints related to the orofacial myofunctional complex is an important step in the evaluation process, as it allows us to understand patients' perceptions concerning orofacial functions. However, clinical practice in Orofacial Motricity indicates that patients do not always have well-defined complaints, which can be associated with a lack of proprioception and also with a lack of awareness of normal patterns of orofacial functions.

In addition to assessing myofunctional complaints, the protocol also includes understanding general health complaints. Regarding the data from this study, it is noted that the highest scores refer to complaints of sleep with snoring, heavy breathing during sleep and the presence of sleep apnea, which is justified by the untreated SDB presented by the patients. Frequent complaints of clenching teeth and bruxism may indicate a condition of sleep and wake bruxism, and studies indicate that these conditions may occur concomitantly with OSA, since they share some risk factors^(13)^.

As for breathing complaints, although some patients occasionally present with rhinitis and nasal obstruction, clinical evaluation did not indicate the presence of oral breathing. As it is a multifactorial condition, different breathing modes may be associated with SDB^(14)^.

About chewing, the main complaint presented was the use of only one side during chewing, which was evidenced and confirmed by clinical evaluation. Alterations in the chewing pattern with unilateral predominance in patients with SDB have already been demonstrated in the literature^(5,6,9)^. In addition to the altered chewing pattern, the analysis also found increased chewing speed and inefficient chewing. Chewing is a complex function related to the eating process. In order for it to be performed in a coordinated and bilateral manner, the facial and masticatory muscles need to work in an integrated manner^(15,16)^. In patients with SDB, damage to the orofacial muscles, characterized mainly by hypotonia and difficulty in performing orofacial movements, is observed^(5,6,9)^.

Concerning swallowing, there were reports of choking, residues after swallowing and difficulty swallowing, while clinical analysis showed excessive contraction of the perioral muscles and associated head movement during swallowing, and the presence of residues in the oral cavity. Such characteristics are reported in other studies^(5,6,9)^, and are mainly justified by the aforementioned lack of coordination and hypotonia of the orofacial muscles present in these patients, with emphasis on changes in tongue function. It is known that the tongue plays a fundamental role in swallowing, especially with regard to the formation of the food bolus and ejection into the pharynx^(17)^. In this study, tongue hypotonia was frequent in the clinical evaluation, as well as changes in orofacial movements.

Although the statistical analysis did not indicate a significant correlation, it was observed that swallowing was the function that presented the most changes in the clinical evaluation, while in the Clinical History it was the function that patients reported the least symptoms. Swallowing in patients with OSA has been studied in several studies cited in a literature review, which show that this population may present signs of swallowing disorders, mainly with the presence of premature posterior leakage and residues^(18)^. Such changes are usually linked to the pathophysiological process of OSA, which leads to episodes of hypoxia and hypercapnia and neuromuscular changes in the tissues of the upper airway, including the pharynx. Furthermore, studies show that changes in swallowing are underdiagnosed in the clinical context, which is a fact that highlights the need for greater focus on this issue^(19)^.

Finally, the statistical analysis identified a significant correlation only between signs and symptoms related to chewing function, being inversely proportional. This data indicates that the signs of changes were not equivalent to the symptoms reported by the patients.

As previously mentioned, clinical complaints are important for defining a personalized therapeutic plan, allowing the professional to assess the symptoms. However, in the context of Orofacial Motricity, in general, many patients do not attribute certain characteristics as alterations, such as individuals who are unaware of the harm caused by unilateral chewing, choking during eating or breathing through the oronasal system. When asked if they have complaints regarding chewing, swallowing and breathing, it is common for these patients to deny it – even in the presence of the alteration, as they are not aware that such characteristics are considered dysfunctions.

The rehabilitation of orofacial functions of breathing, chewing and swallowing is a fundamental part of speech-language pathology therapy intervention in SDB. Although the indication and performance of oropharyngeal exercises are important for the treatment of the disorder^(4)^, working with these functions promotes the balance of the stomatognathic system. If an adequate intervention is not performed, the functions will be performed in an adapted manner and with muscular and postural compensations, which may lead to muscular imbalances and impair the stability of the treatment.

In this context, to evaluate, identify and diagnose the changes in orofacial functions, even in the absence of complaints, is the role and competence of the speech-language pathology therapist, and the participation of these professionals in the decision on the best therapeutic approaches in SDB is essential.

Furthermore, it is also important to note that simple questions such as “Do you have difficulty breathing/chewing/swallowing?” may not be effective in identifying symptoms, and the speech-language pathologist must ask more assertive questions in search of more details and clinical information, preferably based on structured protocols, such as the one used in this study^(10)^. Additionally, it is equally important that the clinical evaluation be conducted in a standardized manner, and that the data obtained be considered for the development of the therapeutic plan. The feedback of the findings to the patient must be conducted objectively by the speech-language pathologist, pointing out the changes found and justifying the therapeutic goals that will be addressed.

Awareness about the proper performance of orofacial functions and the importance of balancing the stomatognathic system should be further promoted and addressed through health promotion actions, allowing individuals to be able to identify deviations from normality in themselves, facilitating the search for integrated treatment^(20)^.

This study has some limitations, mainly regarding the small number of participants, as it was composed of a heterogeneous group and without a control group. It is suggested that further research be carried out with larger samples and comparing groups in relation to sex and severity of SDB.

It is expected that the data obtained in this study will contribute to expanding the discussion about the role of the speech-language pathology therapist in the treatment of SDB, with an emphasis on an objective clinical assessment and individualized treatment.

## CONCLUSION

In the sample studied, a moderate negative correlation was found between masticatory signs and symptoms in adults with SDB, indicating that the greater the presence of clinical signs of alteration in this function, the lower the occurrence of symptoms. No correlations were found between signs and symptoms for breathing and swallowing functions, as well as for the parameters of signs of altered mobility and orofacial tonicity, when correlated with items from the clinical history. These findings point to a non-direct relationship or absence of relationship between signs and symptoms of orofacial myofunctional disorders in this population.
