# Supplementary material for: Orofacial myofunctional signs and symptoms in adults with sleep breathing disorder: is there a correlation?
Source: Codas. 2025 Apr 28;37(3):e20240033. doi: 10.1590/2317-1782/e20240033en (PMC12061340; doi:10.1590/2317-1782/e20240033en)
Supplement: Translation

# Translation: Sinais e sintomas miofuncionais orofaciais em adultos com distúrbio respiratório do sono: existe correlação?

**DOI:** 10.1590/2317-1782/e20240033pt

**Authors:** Gabriele Ramos de Luccas, Raphaela Godoi Abu Halawa, Carlos Henrique Ferreira Martins, Giédre Berretin-Felix

**Affiliations:** 1 Departamento de Fonoaudiologia, Faculdade de Odontologia de Bauru – FOB, Universidade de São Paulo – USP – Bauru (SP), Brasil.

**Keywords:** Apneia Obstrutiva do Sono, Ronco, Fonoaudiologia, Terapia Miofuncional, Língua, Sistema Estomatognático

## Abstract

**Objetivo:**

verificar se sintomas miofuncionais orofaciais se relacionam com achados da avaliação clínica miofuncional orofacial em adultos com distúrbio respiratório do sono (DRS).

**Método:**

estudo observacional; 15 adultos com média de idade de 43 anos e com diagnóstico de ronco primário ou AOS por polissonografia; submetidos ao Protocolo de Avaliação Miofuncional Orofacial MBGR, incluindo o Protocolo de História Clínica para levantamento dos sintomas, e o Protocolo de Exame Clínico para a identificação dos sinais, considerando as provas de mobilidade de lábios, língua, véu palatino e mandíbula; tonicidade de lábios, língua, bochechas e mento; modo respiratório; mastigação; deglutição de sólido e líquido. Para a correlação entre sinais e sintomas foi utilizado o Coeficiente de Correlação de Spearman, considerando estatisticamente significante p<0,05.

**Resultados:**

os principais sintomas miofuncionais orofaciais foram relacionados à mastigação (uso de apenas um lado durante a mastigação e necessidade de ingestão de líquidos), e à deglutição (engasgos e resíduos após a deglutição). Na avaliação miofuncional orofacial houve maior frequência de alteração para tonicidade de língua; mobilidade de lábios; padrão mastigatório unilateral com velocidade aumentada e ineficiência mastigatória; deglutição com contração excessiva da musculatura perioral, movimento de cabeça associado e resíduos na cavidade oral. A correlação entre os escores de sinais e sintomas miofuncionais orofaciais apontou significância apenas entre os aspectos relacionados à função de mastigação, sendo moderada e inversamente proporcional.

**Conclusão:**

foi encontrada correlação moderada negativa entre sinais e sintomas mastigatórios em adultos com DRS, não tendo sido observada correlação para as funções de respiração e deglutição.

## INTRODUÇÃO

Os distúrbios respiratórios do sono (DRS) são condições multifatoriais e crônicas com consequências relacionadas à qualidade do sono, sintomas diurnos de sonolência e prejuízos a atividades diárias^(1)^. Os DRS mais comuns são o ronco primário e os quadros de apneia obstrutiva do sono (AOS), que se referem a episódios de obstrução da via aérea superior durante o sono. Principalmente nos casos de AOS, há associação com diversas comorbidades, a exemplo de doenças cardiovasculares e metabólicas^(2)^.

Nesse sentido, o diagnóstico precoce é fundamental e obtido, principalmente, por exames de polissonografia. O tratamento é interdisciplinar e envolve a participação de médicos, fisioterapeutas, odontólogos, fonoaudiólogos, dentre outros profissionais, para discussão e definição das melhores estratégias terapêuticas, que incluem o tratamento isolado ou combinado de perda de peso, uso de *Continuous Positive Airway Pressure* (CPAP), aparelho intraoral de avanço mandibular, cirurgias^(3)^, terapia miofuncional orofacial (TMO)^(4)^, entre outras abordagens.

Os estudos sobre os resultados da TMO para casos de ronco primário e AOS começaram a ser desenvolvidos no Brasil a partir de 2009, com propostas de tratamento que incluíam exercícios orofaríngeos e intervenção nas funções orofaciais^(5)^. De acordo com a literatura, atualmente há dados que demonstram a efetividade desse tratamento para reduzir sinais e sintomas de AOS e intensidade e frequência do ronco, além de melhora nos aspectos miofuncionais orofaciais^(6-8)^.

A identificação de alterações miofuncionais orofaciais apresentadas por pacientes com AOS foi o ponto de partida inicial para o desenvolvimento das propostas de tratamento no âmbito da especialidade de Motricidade Orofacial, em contexto fonoaudiológico. De maneira geral, esses indivíduos possuem tendência em apresentar alongamento do palato mole; aumento na musculatura lingual; flacidez da úvula, arcopalatoglosso, musculatura da mímica facial, parede lateral da faringe, músculos bucinador, orbicular da boca e suprahioideos; pouca mobilidade de face; alterações na mastigação e deglutição; e respiração oronasal^(9)^.

O planejamento terapêutico miofuncional orofacial para casos de DRS é baseado em evidências científicas e personalizado clinicamente para cada paciente, considerando suas particularidades e principais alterações e limitações miofuncionais. Nesse sentido, o processo de avaliação miofuncional orofacial é essencial, pois permite a análise e interpretação de importantes aspectos estruturais, musculares e funcionais que devem ser levados em consideração nos objetivos e metas terapêuticas.

Protocolos clínicos de avaliação miofuncional orofacial são aplicados nas pesquisas em TMO para casos de DRS com o objetivo de análise de resultados pré e pós intervenção e, embora abordem as características miofuncionais orofaciais dessa população^(10,11)^, ainda não há estudos mais detalhados sobre essas características, principalmente que correlacionem dados clínicos com sintomas e queixas dos pacientes.

Os sintomas miofuncionais orofaciais podem incluir queixas relacionadas às funções orofaciais de respiração, mastigação, deglutição e fala e, ainda, apontar problemas relacionados à estética facial. No contexto clínico, a identificação de sintomas no início do processo terapêutico é fundamental para traçar as prioridades do tratamento e alinhar expectativas com os pacientes. Da mesma forma, a identificação de sinais clínicos é importante para direcionamento e seleção de exercícios e estratégias terapêuticas. A relação entre a presença de sinais e sintomas é comum, porém podem haver casos em que o paciente não possui autopercepção de suas alterações miofuncionais, o que resulta na ausência de queixas e sintomas.

A obtenção de dados sobre os principais sinais e sintomas miofuncionais orofaciais apresentados por pacientes com DRS, bem como a correlação entre essas informações, pode auxiliar no direcionamento do olhar do fonoaudiólogo clínico que atende essa população. Tendo em vista que tais informações podem aprimorar o raciocínio teórico-prático, o presente trabalho tem por objetivo verificar se sintomas miofuncionais orofaciais se relacionam com achados da avaliação clínica miofuncional orofacial em adultos com DRS.

## MÉTODO

Trata-se de um estudo observacional, previamente aprovado pelo Comitê de Ética em Pesquisa da Faculdade de Odontologia da Universidade de São Paulo (FOB/USP) (CAAE: 53781916.7.0000.5417), pelo parecer Nº 3.284.811. Todos os participantes da pesquisa foram incluídos no projeto mediante assinatura em Termo de Consentimento Livre e Esclarecido (TCLE).

Foram selecionados 15 adultos com média de idade de 43 anos (desvio padrão: 10,2), com diagnóstico de ronco primário ou AOS, evidenciado por meio de exame de polissonografia laudado por um médico especialista em distúrbios do sono. Os participantes foram recrutados a partir de encaminhamentos médicos realizados para a Clínica de Fonoaudiologia da FOB/USP. Os procedimentos de coleta e análise de dados também foram realizados na respectiva clínica, no período de julho a dezembro de 2016, por uma única pesquisadora.

Foram incluídos na pesquisa os participantes que apresentaram idade entre 19 e 59 anos, de ambos os gêneros, e com diagnóstico de AOS de diferentes graus ou ronco primário comprovados por meio do exame de polissonografia. Foram excluídos adultos que apresentaram histórico de: doenças pulmonares, tais como insuficiência respiratória aguda, embolia pulmonar, asma, necessidade de suporte ventilatório, pós operados de cirurgias torácicas; alterações neurológicas; doenças oncológicas de cabeça e pescoço; fumantes atualmente; terapia fonoaudiológica prévia na área de motricidade orofacial ou disfagia orofaríngea e realização de exame mais atual de polissonografia realizado há um período superior de seis meses.

Os critérios de inclusão e exclusão foram aplicados a partir de entrevista inicial para coleta da história clínica e da avaliação miofuncional orofacial prévia a partir de protocolo de exame clínico^(10)^, considerando as provas de análise de padrão facial e análise intraoral para exclusão de indivíduos com alteração de frênulo lingual, má oclusão, e discrepâncias maxilomandibulares.

Após a seleção da amostra da pesquisa, todos os participantes do estudo foram submetidos aos mesmos procedimentos de avaliação e análise.

### Protocolo de Avaliação Miofuncional Orofacial MBGR

O Protocolo de Avaliação Miofuncional Orofacial MBGR é um instrumento de avaliação com escores composto por história clínica e exame clínico, desenvolvido para os profissionais da especialidade fonoaudiológica de Motricidade Orofacial^(10)^. Para aplicação deste protocolo em prática ou pesquisa clínica, é necessário treinamento adequado para uma correta coleta e interpretação dos dados.

A pesquisadora responsável pelo projeto realizou cursos e sessões práticas para aplicação do instrumento de forma padronizada em pacientes modelo, com condução e supervisão direta de uma das autoras do protocolo. A pesquisadora realizou todos os procedimentos nos pacientes desta pesquisa.

### História Clínica – MBGR

O protocolo para levantamento de dados de história clínica é composto por questões que buscam a identificação de queixas e sintomas relacionados a problemas de saúde, tratamentos prévios, sono, problemas respiratórios, mastigação, deglutição, alimentação atual, hábitos orais deletérios, e também aspectos envolvendo a comunicação, fala, audição, voz e escolaridade. Como forma de facilitar a tabulação e correlação de dados, os pesquisadores estipularam uma pontuação para cada item do protocolo conforme a frequência do sintoma apresentado pelo paciente, sendo: 0 (ausência de sintoma), 1 (presença esporádica de sintomas), e 2 (presença frequente de sintomas). Sendo assim, quanto maior o escore, maior a é a queixa do paciente quanto aquele aspecto.

A pesquisadora realizou uma entrevista com os pacientes em local confortável e silencioso para a coleta e registro das informações fornecidas.

### Exame clínico miofuncional orofacial

Além da história clínica, o protocolo contém uma proposta de avaliação dos aspectos miofuncionais orofaciais denominado Avaliação Miofuncional Orofacial MBGR, que atribui escores para classificar as alterações encontradas. Nesta amostra, o protocolo contemplou os aspectos descritos a seguir, sendo que para cada item foi atribuído um valor em que o número 0 representa ausência de alteração e números superiores presença de alterações, sendo que, quanto maior o valor total, maior o nível de alteração. Foram realizados os seguintes itens do protocolo com os pacientes:

Mobilidade de lábios, língua, véu palatino e mandíbula: foi feita a solicitação verbal para as tarefas motoras, as quais foram filmadas para análise posterior. Para avaliar a mobilidade dos lábios, foi feita análise dos movimentos de protrair fechados e abertos, retrair fechados e abertos, protrair fechados à direita e esquerda e estalar protraídos e retraídos. Na avaliação da língua foram avaliados os movimentos de protrair e retrair, elevar o ápice na papila incisiva, pontos cardeais com ápice da língua, tocar internamente as bochechas direita e esquerda, estalar o ápice e o corpo, sugar a língua no palato e vibrar. O véu palatino foi avaliado pela da emissão da vogal [a] repetidamente e a mandíbula foi analisada por meio da abertura e fechamento da boca, lateralidade à direita e esquerda, observando-se a presença de desvio e/ou dor;
Tonicidade de lábios, língua, bochechas e mento: foi feita a palpação dos músculos para que eles pudessem ser classificados como normais, hipotônicos ou hipertônicos;
Modo respiratório: foi realizada a classificação em nasal, oronasal ou oral. O espelho milimetrado de Glatzel foi utilizado para fazer-se a verificação do fluxo de ar nasal do paciente ao chegar e após a limpeza. Foi observada a simetria do fluxo entre as narinas, além de avaliar-se a possibilidade de uso nasal com o teste de água na boca, onde era solicitado que o paciente permanecesse com o líquido na cavidade oral por dois minutos ou mais para ser considerado adequado.
Mastigação: foi avaliada a mastigação habitual de três porções de bolacha *wafer,* sendo observada a incisão, trituração, tempo, padrão mastigatório, fechamento labial, velocidade, ruídos e contrações musculares atípicas.
Deglutição: foi avaliada a deglutição habitual e dirigida de líquido (água em temperatura ambiente ofertada em copo transparente) e sólido (bolacha *wafer*). Foi observado o fechamento labial, postura de língua, contenção, contrações musculares atípicas, movimento de cabeça, ruídos, coordenação e resíduos após deglutir.

### Análise de dados

Utilizou-se análise descritiva para apresentação de dados de caracterização da amostra. Para a correlação entre sinais e sintomas foi utilizado o Coeficiente de Correlação de Spearman, considerando estatisticamente significante p<0,05. Considerou-se para correlação os escores totais de sinais e sintomas, bem como itens específicos do Protocolo de História Clínica (problemas de saúde; problemas respiratórios; sono, hábitos de mordida; mastigação; deglutição) que foram correlacionados com itens do Protocolo de Avaliação Miofuncional Orofacial (mobilidade, tonicidade, respiração, mastigação, deglutição). Os itens de sintomas de sono e hábitos de mordida foram correlacionados apenas com os sinais das funções orofaciais (respiração, mastigação e deglutição).

A força de correlação obtida foi classificada a partir da referência de um estudo prévio^(12)^, em que r=0,10 até 0,30 é considerado fraca; r=0,40 até 0,60 é moderada; e =0,70 até 1 é forte.

## RESULTADOS

Inicialmente, 38 indivíduos com diagnóstico de DRS foram considerados para essa pesquisa. Destes, 14 recusaram-se a participar devido a impossibilidade de comparecer para os atendimentos; dois não compareceram às consultas agendadas para coleta dos dados; e sete não atenderam aos critérios de inclusão e exclusão por apresentarem anquiloglossia (n=4), padrão facial II (n=2) e ausência de elementos dentários (n=1).

Na Tabela 1 encontram-se os dados de caracterização dos participantes, tais como idade, sexo, Índice de Apneia e Hipopneia (IAH) e escore total de sintomas e sinais obtidos pelo Protocolo MBGR. A amostra foi constituída por 15 participantes, sendo a maioria do sexo masculino (73,3%), com faixa de idade predominante entre 40 e 50 anos e com diversos graus de severidade de AOS. A pontuação de sinais e sintomas variaram entre os participantes, sendo 18 e 45 os escores mínimo e máximo para sintomas e 15 e 39 para sinais, respectivamente.

**Tabela 1:** Caracterização da amostra quanto ao sexo, idade, Índice de Apneia e Hipopneia (IAH) e escore total de sintomas e sinais obtidos pelo Protocolo MBGR

Paciente	Sexo	Idade	IAH	Escore total de sintomas		Escore total de sinais
				Mínimo: 0		Mínimo: 0
				Máximo: 216		Máximo: 175
1	F	38	3,3	41	31	
2	M	55	67,7	28	33	
3	M	43	2,8	35	21	
4	M	37	24,3	30	15	
5	F	22	6,8	42	17	
6	M	34	5,8	36	27	
7	M	59	19,4	29	28	
8	F	50	7,0	29	39	
9	M	43	57,4	35	26	
10	M	42	14,7	38	26	
11	M	49	4,4	43	34	
12	M	56	14	25	35	
13	F	31	6,8	59	21	
14	M	34	7,9	45	16	
15	M	49	29,1	18	22	

**Legenda: F =** Feminino; **M =** Masculino; **IAH =** Índice de Apneia e Hipopneia

Na Tabela 2 encontra-se para cada participante os escores obtidos referentes aos problemas de saúde, problemas respiratórios, mastigação, deglutição, sono, e hábitos de mordida. Nota-se que os maiores escores referem-se às queixas de sono, sendo que os pacientes referiram, com mais frequência, à presença do ronco (n=14), ressonar (n=11) e da apneia do sono (n=13).

**Tabela 2:** Aspectos gerais da amostra quanto às queixas relacionadas a problemas de saúde, problemas respiratórios, mastigação, deglutição, sono, e hábitos de mordida

Paciente	Problemas de saúde	Problemas respiratórios	Mastigação	Deglutição	Sono	Hábitos de mordida
	Mínimo= 0	Mínimo= 0	Mínimo= 0	Mínimo= 0	Mínimo= 0	Mínimo= 0
	Máximo= 5	Máximo= 13	Máximo= 16	Máximo= 22	Máximo= 22	Máximo= 5
1	0	6	3	4	7	2
2	2	1	2	0	13	2
3	2	0	1	2	14	0
4	1	1	2	0	15	2
5	2	4	2	0	17	2
6	1	4	1	1	8	3
7	2	3	2	0	14	2
8	2	5	0	1	9	0
9	1	3	7	0	11	0
10	0	7	1	1	10	1
11	1	1	2	0	12	3
12	1	3	3	1	7	2
13	4	1	3	3	17	3
14	1	6	3	2	10	3
15	1	4	0	3	1	1

Os problemas de saúde relatados foram do tipo ortopédico (n=7), metabólico (n=7), digestivo (n=2) e hormonal (n=2), enquanto que os respiratórios se referem à obstrução nasal (n=8), rinite (n=8), coriza (n=7), halitose (n=5) e prurido nasal (n=5). Em relação aos hábitos de mordida, estes apontaram, em sua maioria, apertamento dentário (n=10), bruxismo (n=7) e hábito de morder a mucosa (n=5). Com relação à mastigação, as principais queixas apresentadas foram referentes ao uso de apenas um lado durante a mastigação (n= 11) e a necessidade de ingestão de líquidos durante as refeições (n= 6). No que se refere à deglutição, a queixa apresentada foi referente a engasgos (n=6), resíduos após a deglutição (n=3) e dificuldade para deglutir (n=1).

A Tabela 3 apresenta os resultados individuais da avaliação miofuncional orofacial realizada pelo protocolo MBGR, em que se encontram os escores obtidos para tonicidade (lábios, língua, bochechas e mento), mobilidade (lábios, língua, véu palatino e mandíbula), respiração, mastigação e deglutição.

**Tabela 3:** Apresentação dos escores individuais obtidos para os participantes do estudo nas provas de tonicidade, mobilidade, respiração, mastigação e deglutição na avaliação clínica

Paciente	Tonicidade	Mobilidade	Respiração	Mastigação	Deglutição
N=15	Mínimo= 0 Máximo= 6	Mínimo= 0 Máximo= 68	Mínimo= 0 Máximo= 4	Mínimo= 0 Máximo= 10	Mínimo= 0 Máximo= 36
1	5	14	0	2	9
2	2	18	0	2	10
3	2	4	0	5	9
4	2	5	0	2	6
5	5	7	0	2	2
6	4	14	0	3	5
7	4	13	0	3	7
8	3	26	0	3	6
9	4	17	0	2	2
10	4	7	0	3	11
11	3	15	0	1	14
12	3	19	0	2	10
13	1	9	0	2	8
14	1	6	0	2	7
15	5	7	0	2	7

Em relação à tonicidade orofacial, houve maior frequência de hipotonia para língua (n=12), seguido por lábios (n=9) e bochechas (n=7), e nenhum participante apresentou tônus aumentado.

No que se refere à mobilidade, a alteração na mobilidade de lábios foi a mais frequente (n=11). Para todas as provas de mobilidade os pacientes apresentaram pontuação indicativa de normalidade ou alteração, não tendo sido encontrado incapacidade de realizar algum movimento solicitado. Notou-se também, com frequência, que a maioria dos pacientes apresentava movimentos associados da musculatura cervical ao executar as provas de mobilidade de língua e mandíbula.

Em relação à função respiratória, todos os pacientes apresentaram modo respiratório nasal (n=15). Na função mastigatória, as alterações encontradas referem-se ao padrão mastigatório unilateral (n=7), velocidade aumentada (n=9) e ineficiência mastigatória (n=8). Por fim, na deglutição, a maioria das alterações estão relacionadas a contração excessiva da musculatura perioral (n=11), movimento de cabeça associado (n=4) e presença de resíduos na cavidade oral (n=5).

A Tabela 4 demonstra que houve correlação significante entre os escores de sinais e sintomas miofuncionais orofaciais apenas entre os aspectos relacionados à função de mastigação (p=0,034), sendo inversamente proporcional e com força de correlação moderada (r=-0,548).

**Tabela 4:** Apresentação das correlações obtidas entre sinais e sintomas das funções de respiração, mastigação e deglutição

Escore total de sintomas	Escore total de sinais	Valor r	Valor p
Respiração	Respiração	-0,046	0,870
Mastigação	Mastigação	-0,548	0,034*
Deglutição	Deglutição	0,191	0,496

* p<0,05

## DISCUSSÃO

As características miofuncionais orofaciais de pacientes com DRS direcionam os fonoaudiólogos na seleção de indivíduos elegíveis para tratamento, além de serem fundamentais para escolha e seleção de metas e estratégias terapêuticas personalizadas para cada caso. O Protocolo de Avaliação Miofuncional Orofacial MBGR é utilizado com frequência na avaliação clínica de pacientes com DRS no contexto dos ensaios clínicos randomizados^(5,6)^, além de ser utilizado com frequência na rotina clínica dos fonoaudiólogos, sendo composto por levantamento de queixas clínicas e avaliação miofuncional orofacial^(10)^.

O levantamento de queixas clínicas relacionadas ao complexo miofuncional orofacial é uma etapa importante do processo avaliativo, já que permite compreender as percepções dos pacientes em relação às funções orofaciais. Porém, a prática clínica em Motricidade Orofacial indica que nem sempre os pacientes possuem queixas bem definidas, o que podemos associar a uma falta de propriocepção e também à ausência de conscientização sobre padrões de normalidade das funções orofaciais.

Além do levantamento das queixas miofuncionais, o protocolo utilizado também prevê a compreensão de queixas de saúde geral. Em relação aos dados dessa pesquisa, nota-se que os maiores escores referem-se às queixas de sono com ronco, ressonar e presença de apneia do sono, o que se justifica pelo quadro de DRS não tratada apresentado pelos pacientes. As queixas frequentes de apertamento dentário e bruxismo podem indicar um quadro de bruxismo do sono e de vigília, e estudos indicam que essas condições podem acontecer de maneira concomitante aos quadros de AOS já que compartilham alguns fatores de risco^(13)^.

No que se refere a queixas respiratórias, embora alguns pacientes apresentem quadros de rinite e obstrução nasal eventualmente, a avaliação clínica não indicou presença de respiração oral. Sendo uma condição multifatorial, diferentes modos respiratórios podem estar associados aos quadros de DRS^(14)^.

Com relação à mastigação, a maior queixa apresentada foi o uso de apenas um lado durante a mastigação, o que foi evidenciado e confirmado pela avaliação clínica. Alterações no padrão mastigatório com predomínio unilateral em pacientes com DRS já foi evidenciado em literatura^(5,6,9)^. Além do padrão mastigatório alterado, a análise também verificou a presença de velocidade mastigatória aumentada e ineficiência mastigatória. A mastigação é uma função complexa relacionada ao processo alimentar. Para que seja realizada de maneira coordenada e de forma bilateral, os músculos faciais e mastigatórios precisam trabalhar de maneira integrada^(15,16)^. Em pacientes com DRS observa-se prejuízos na musculatura orofacial, caracterizados principalmente por hipotonia e dificuldade na realização de movimentos orofaciais^(5,6,9)^.

No que se refere à deglutição, houveram relatos de engasgos, resíduos após a deglutição e dificuldade para deglutir, enquanto que a análise clínica evidenciou contração excessiva da musculatura perioral e movimento de cabeça associado durante a deglutição, e presença de resíduos na cavidade oral. Tais características são relatadas em outros estudos^(5,6,9)^, e são justificadas principalmente pela já citada incoordenação e hipotonia dos músculos orofaciais presentes nesses pacientes, com destaque para alteração na função de língua. Sabe-se que a língua exerce papel fundamental na deglutição, principalmente no que se refere a formação do bolo alimentar e ejeção para a faringe^(17)^. Nesse estudo, a hipotonia de língua foi frequente na avaliação clínica, bem como a alteração nos movimentos orofaciais.

Embora a análise estatística não tenha apontado correlação significativa, observou-se que deglutição foi a função que mais apresentou alteração na avaliação clínica, enquanto que, na História Clínica, foi a função que os pacientes menos relataram sintomas. A deglutição em pacientes com AOS tem sido objeto de estudo em diversos trabalhos citados em uma revisão de literatura, os quais evidenciam que essa população pode apresentar sinais de alteração na deglutição, principalmente com presença de escape posterior prematuro e resíduos^(18)^. Tais alterações normalmente são vinculadas ao processo fisiopatológico da AOS, o qual acarreta episódios de hipóxia e hipercapnia e alterações neuromusculares aos tecidos da via aérea superior, incluindo a faringe. Além disso, os estudos evidenciam que alterações na deglutição são subdiagnosticadas no contexto clínico, sendo um dado que evidencia a necessidade de maior foco nessa questão^(19)^.

Por fim, a análise estatística identificou correlação significativa apenas entre sinais e sintomas relacionados à função de mastigação, sendo inversamente proporcional. Esse dado indica que os sinais de alterações não foram equivalentes aos sintomas relatados pelos pacientes.

Assim como citado anteriormente, as queixas clínicas são importantes para definição do plano terapêutico personalizado, permitindo uma valorização dos sintomas por parte do profissional. Porém, no contexto da Motricidade Orofacial, de maneira geral, diversos pacientes não atribuem determinadas características como alterações, a exemplo de indivíduos que não tem conhecimento sobre os prejuízos da mastigação unilateral, da presença de engasgos durante a alimentação ou da respiração realizada de maneira oronasal. Ao serem questionados se possuem queixas em relação a mastigação, deglutição e respiração, é comum que esses pacientes neguem – mesmo na presença da alteração, por não terem conhecimento que tais características são consideradas disfunções.

A reabilitação das funções orofaciais de respiração, mastigação e deglutição é parte fundamental da intervenção fonoaudiológica em DRS. Embora a indicação e realização de exercícios orofaríngeos sejam importantes para o tratamento do distúrbio^(4)^, o trabalho com tais funções promove o equilíbrio do sistema estomatognático. Caso não seja realizada uma intervenção adequada, as funções serão realizadas de maneira adaptada e com compensações musculares e posturais, o que poderá acarretar desequilíbrios musculares e prejudicar a estabilidade do tratamento.

Nesse contexto, a avaliação, identificação e diagnóstico de alterações nas funções orofaciais mesmo na ausência de queixas é papel e competência do fonoaudiólogo, sendo primordial a participação desses profissionais na decisão sobre as melhores condutas terapêuticas em DRS.

Além disso, ressalta-se, também, que perguntas simples como *“Você tem dificuldade para respirar/mastigar/engolir?”* podem não ser efetivas na identificação de sintomas, sendo necessário que o fonoaudiólogo conduza perguntas mais assertivas em busca de maiores detalhes e informações clínicas, preferencialmente com base em protocolos estruturados, a exemplo do utilizado nesse estudo^(10)^. Adicionalmente, é igualmente importante que a avaliação clínica seja conduzida de maneira padronizada, e que os dados obtidos sejam considerados para elaboração do planejamento terapêutico. A devolutiva dos achados ao paciente deve ser conduzida com objetividade pelo fonoaudiólogo, apontando as alterações encontradas e justificando as metas terapêuticas que serão abordadas.

A conscientização sobre a adequada realização das funções orofaciais e a importância do equilíbrio do sistema estomatognático deve ser mais promovida e abordada por meio de ações de promoção de saúde, permitindo que os indivíduos sejam capazes de identificar em si desvios da normalidade e facilitando a procura por um tratamento integrado^(20)^.

O presente estudo possui algumas limitações, principalmente no que se refere ao número pequeno de participantes, por ser composto por um grupo heterogêneo e sem grupo controle. Sugere-se que novas pesquisas sejam realizadas com amostras maiores e comparando grupos em relação ao sexo e gravidade do DRS.

Espera-se que os dados obtidos nessa pesquisa colaborem para a ampliação da discussão acerca do papel do fonoaudiólogo no tratamento dos DRS, com valorização de uma avaliação clínica objetiva e um tratamento individualizado.

## CONCLUSÃO

Conclui-se que, na amostra estudada na presente pesquisa, foi encontrada correlação negativa moderada entre sinais e sintomas mastigatórios em adultos com DRS, indicando que quanto maior a presença de sinais clínicos de alteração nessa função, menor é a ocorrência de sintomas. Não foram encontradas correlações entre sinais e sintomas para as funções de respiração e deglutição, bem como para os parâmetros de sinais de alteração de mobilidade e tonicidade orofacial quando correlacionados com itens da história clínica. Tais achados apontam para uma relação não direta ou ausência de relação entre sinais e sintomas de distúrbios miofuncionais orofaciais nessa população.
